# A New Logistic Dynamic Particle Swarm Optimization Algorithm Based on Random Topology

**DOI:** 10.1155/2013/409167

**Published:** 2013-05-30

**Authors:** Qingjian Ni, Jianming Deng

**Affiliations:** ^1^School of Computer Science and Engineering, Southeast University, Nanjing 211189, China; ^2^Provincial Key Laboratory for Computer Information Processing Technology, Soochow University, Suzhou 215006, China

## Abstract

Population topology of particle swarm optimization (PSO) will directly affect the dissemination of optimal information during the evolutionary process and will have a significant impact on the performance of PSO. Classic static population topologies are usually used in PSO, such as fully connected topology, ring topology, star topology, and square topology. In this paper, the performance of PSO with the proposed random topologies is analyzed, and the relationship between population topology and the performance of PSO is also explored from the perspective of graph theory characteristics in population topologies. Further, in a relatively new PSO variant which named logistic dynamic particle optimization, an extensive simulation study is presented to discuss the effectiveness of the random topology and the design strategies of population topology. Finally, the experimental data are analyzed and discussed. And about the design and use of population topology on PSO, some useful conclusions are proposed which can provide a basis for further discussion and research.

## 1. Introduction

Particle swarm optimization (briefed as PSO) is a kind of bionic evolutionary algorithm which rooted in imitation of behavioral mechanisms in populations such as birds and fish stocks and has been widely used in engineering field as optimization method [1–3].

In the PSO algorithms, the particles evolve according to their own experience and the experience of the neighborhood particles. During the evolutionary process, particles identify their own neighborhood according to the population topology and then learn from each other and update the positions of the particles. Therefore, population topology determines the form of information sharing among particles and thus has a very important impact on the solving performance of PSO algorithms. Therefore, it is important to explore the population topologies of PSO algorithms. This will produce a deep understanding of the working mechanism of PSO algorithms and thus improve the solving performance.

In the PSO algorithms, the most common used static population topologies are the fully connected topology (Gbest model) and the ring topology (lbest model) which are also first proposed [4]. Since then, researchers have proposed different population topologies in succession. Kennedy carried out a preliminary analysis of four static population topologies [5]. Suganthan adjusted the neighborhood structure of particles through calculating distances between particles in the evolutionary process [6]. Mendes et al. detailly analyzed the relationship between the population topology and a class of PSO variant [7–9]. Clerc initially attempted to adopt the random topology [10]. However, these studies concerned population topologies paid more attention to the static classic population topology, and research of random population topologies is relatively small. As the population topology directly affect the exchange of information between the particles, it is necessary to design the suitable population topology according to the characteristics of different types of applications. Therefore, it is necessary to explore the population topologies in depth from a theoretical and experimental point of view.

In this paper, in a relatively new PSO variant which named logistic dynamic particle optimization, we analyze the linkages between population topologies and the performance of PSO algorithms from graph theory and experimental point of view. The rest of the paper is organized as follows. In Section 2, we describes the PSO variant which are used in the paper.  Section 3 describes the classic population topologies and introduces the proposed random population topologies. In Section 4, we have presented the experimental analysis and comparative performance between the classic and the proposed random population topologies.  Section 5 concludes the paper.

## 2. The PSO Variants

 PSO is a population-based method which is similar to other evolutionary computation methods. The individual in the PSO population is called the particle, and particles generally have the speed and position in most PSO variants.

### 2.1. The Canonical PSO

 Based on the earlier version of PSO, Clerc developed the PSO with the compression factor [11]. This PSO variants have been widely used in practical applications, and the velocity and position update of particles in this variant are as follows:

(1) vid=χ∗[vid+c1∗rand(  )∗(pid−xid)  + c2∗Rand(  )∗(pgd−xid)],

(2) $$\begin{array}{c} \chi =\frac{2}{\left|2-\varphi -\sqrt{\varphi^{2}-4\varphi }\right|,} \end{array}$$

(3) $$\begin{array}{c} x_{id}=x_{id}+v_{id}. \end{array}$$

In (1), *v*_*id*_ is the *d*th dimensional component of particle *i*s velocity attribute, *c*_1_ and *c*_2_ are two positive acceleration factors, rand() and Rand() are random number generating functions between 0 and 1, *x*_*id*_ is the *d*th dimensional component of the particle *i*s position property, *p*_*id*_ is the *d*th dimensional component of the best position that particle *i* obtained, and *p*_*gd*_ is the *d*th dimensional component of the best position that the whole population obtained. In actual use, usually *χ* in (2) is set to 0.729, and *φ* is often set to 4.1.

The right part of the equation1 can be understood as particle's memory, cognitive and social cognition. Velocity of particles is precisely through this three-part interaction effects thereby, position of particles is updated. 

### 2.2. The Dynamic Probabilistic Particle Swarm Optimization

 In the previous PSO variants, particles usually have both velocity attribute and position attribute. Kennedy first proposed a new PSO variant without velocity attribute, which named Gaussian dynamic particle swarm optimization [12]. Ni and Deng carried out a further study on the PSO variants without velocity [13]. This type of algorithm variants can be called dynamic probabilistic particle swarm optimization (briefed as DPPSO). In the DPPSO algorithms, particles have no velocity attributes, and the update of particles' positions is reorganized as follows:

(4) Xi(t+1)=Xi(t)+α∗(Xi(t)−Xi(t−1))+β∗CTi(t)+γ∗Gen(  )∗OTi(t),

(5) $$\begin{array}{c} {\text{CT}}_{id}\left(t\right)=\sum_{k=1}^{K}\frac{P_{kd}}{K-X_{id}\left(t\right)}, \end{array}$$

(6) $$\begin{array}{c} {\text{OT}}_{id}\left(t\right)=\sum_{k=1}^{K}\frac{\left|P_{id}-P_{kd}\right|}{K}. \end{array}$$

In (4), (5), and (6), *t* represents the current evolution generation of particle, *i* is the index number of particle, *k* is the index number of particle's neighborhood, *K* represents the number of particle's neighborhood particles, *P*_*k*_ is the optimum position of the particle's neighborhood that numbered *k*, and *d* is the number of the particles *i*s dimension. CT_*i*_(*t*) is an abbreviation of centralized tendency, which is a *D*-dimensional vector and is determined by the particle's current location and neighborhood particles' optimal positions. OT_*i*_(*t*) is an abbreviation of outlier trend, which is also a *D*-dimensional vector and is determined by the particle's current location and neighborhood particles' optimal positions. In (4), *α*, *β*, and *γ* are generally preferable to positive constants. Gen() is a dynamic probabilistic evolutionary operator which is the random number generator function which satisfies a specific distribution, and this particular distribution may be provided by a Gaussian distribution or a logistic distribution and so forth.

In (4), the calculation of particle's position in new generation is decided jointly by the right four parts. The first part is the memory of particles on the self-position. The second part is the trend of the particles along the previous direction of movement of the “flying.” The third part means the influence of neighborhood particles' experience to the new generation position, this part of the calculations needs neighborhood particles' experience, and this part determines the degree of influence of the neighborhood particles' experience. The part IV reflects the impact of differences in best position between particles on the next generation position.

The performance of DPPSO variants is different when using different dynamic probabilistic evolutionary operator Gen() [13]. DPPSO-Gaussian (briefed as GDPS) has faster convergence speed in the early evolution. DPPSO-Cauchy may get better solutions on certain issues, but the performance is unstable. DPPSO-Logistic (logistic dynamic particle swarm optimization, briefed as LDPSO) still shows good exploration ability in the later evolution. For DPPSO variants, the calculation of CT_*i*_(*t*) and OT_*i*_(*t*) will use the experience of each neighborhood particles which can also be seen from (5) and (6). The optimal information of neighborhood particles could be fully utilized, so the research of population topology in DPPSO variants is more important.

## 3. Population Topologies

### 3.1. The Classic Population Topologies

**Figure 1:**
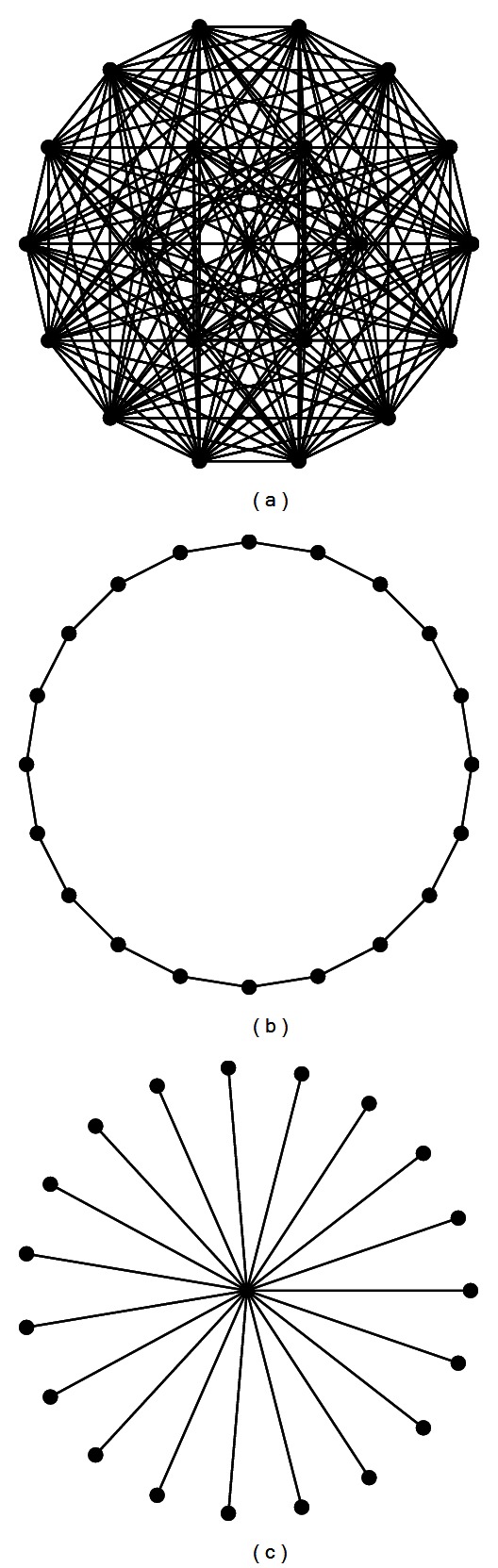
(a) Fully connected topology; (b) ring topology; (c) star topology.

Figure 1 shows the fully connected topology, ring topology, and star topology. Fully connected topology and ring topology are two commonly used topologies which are also called Gbest model and Lbest model. In the fully connected topology, particle's neighborhood contains all particles in the population. And in the evolutionary process, only the particle that obtains the optimal position is considered in the entire population. PSO algorithms with this topology converge very fast but easy to fall into local optimum.

For a ring topology, typically particle's neighborhood includes the particles on both sides of one or a few particles. In this topology, the exchange of information is relatively slow within the population, but once a particle searched for an optimal location, the information eventually will slowly spread to the entire population.

For a star topology, one particle is connected with all the other particles, and other particles only connect with the particle. In addition to a central particle, other particles are independent of each other, the dissemination of information must be passed through the central particle.

However, the information dissemination mechanism of the social groups is not static throughout the whole evolutionary process, and tends to have a certain degree of randomness and dynamic characteristics. Mendes studied random population topology and confirmed that population topology directly affects the execution performance of PSO algorithms [9].

### 3.2. Graph Theory Characteristics of Population Topology

 The population topology of the PSO algorithm can be abstracted into a connected undirected graph, represented by the symbol *G*(*V*, *E*), where *V* is the set of vertices, *E* is the set of edges, and the number of vertices is denoted by *n*. For any two points *u* and *v* in *G*, *d*(*u*, *v*) denotes the distance from *u* to *v*, that is, the length of the shortest path between two points.

**Definition 1 (average degree)**

The degree of the vertex *v* is the number of its adjacent vertices, denoted by *k*_*v*_. Average degree *k* of undirected connected graph is calculated by

(7) $$\begin{array}{c} K=\sum_{v\in V}\frac{k_{v}}{n}. \end{array}$$

**Definition 2 (average clustering coefficient)**

The local clustering coefficient *c*(*v*) of vertex *v*, equals to the number of edges which can be connected between vertices associated with vertex *v*, divided by the maximum number of edges between these vertices. The average clustering coefficient *c* of a graph is the arithmetic mean of the local clustering coefficient of all vertices, which can be calculated by

(8) $$\begin{array}{c} c=\sum_{v\in V}\frac{c\left(v\right)}{n}. \end{array}$$

The average degree of population topology means the average number of particles' neighborhood particles; it represents the degree of socialization of population. A small number of neighborhood particles means that the particle is both difficult to obtain information from the population and difficult to influence other particles. On the contrary, a particle which have large number of neighborhood particles can get a lot of information available in the population, and such a particle has a greater influence in the population. In the common used population topologies, fully connected topology has the largest average degree which is equal to the population size minus 1. The ring topology has the minimum average degree; the average degree of a ring topology such as in Figure 1 is equal to 2. The local clustering coefficient is the ratio of the number of connections between the actual existence and the possible existence, and the average clustering coefficient represents the degree of aggregation of the vertices in a graph which is the average of the local clustering coefficient of all vertices. In this paper, we will analyze the role of the population topology based on the previous graph theory characteristics.

### 3.3. Random Population Topologies

 Clerc initially attempted to proposed a method of random population topology [10]. The basic idea is to generate a random topology by selecting the neighborhood particles randomly for each particle. The concrete steps could be described as Algorithm 1. 

In the resulting matrix *L* of Algorithm 1, *L*(*u*, *v*) = 1 means that the particles *u* and *v* are connected. By this method, a random topology could be generated with an average degree slightly larger than *K*. In the random topology by this method, the distribution of degree is the sum of *S* − 1 independent Bernoulli random variables which is described in [10]

(9) $$\begin{array}{c} \text{prob}\left(Y=n\right)=C_{S-1}^{n-1}{\left(\frac{K}{S}\right)}^{n-1}{\left(1-\frac{K}{S}\right)}^{s-n}. \end{array}$$

In this paper, in order to ensure the connectivity of undirected graph which is corresponding to the generating random topology, we proposed a new generating method of random topology based on Clerc's method. The basic idea is: in selecting the neighborhood particles for each particle if the selection is itself, reselect in order to reduce the probability of particles isolated; after the random population is generated, use the Dijkstra algorithm to compute the distance between the particles if there exist the unconnected particles in the generated topology, then add an edge between these unconnected particles, and retest. The improved random population topology generating method is as Algorithm 2.

## 4. Experiment and Analysis

### 4.1. Experiment Setting

 Two sets of experiments were conducted which used the canonical PSO (briefed as CPSO) and the DPPSO-Logistic, respectively, that are described in Section 2. For CPSO, set *c*_1_ = *c*_2_ = 2.05, *χ* = 0.7298. For DPPSO-Logistic, set *α* = 0.729, *β* = 2.187, and *γ* = 0.5.

The algorithms were used to solve five benchmark functions, which is defined in Table 1. These functions consist of Ackley, Schwefel, Schaffer's F6, Rastrigin, and Sphere.  Table 2 shows the settings of these functions.

In the experiment, the population size is set to 20, in addition to the the Schaffer's F6 function of the dimension 2, the remaining functions are carried out in the case of 30-dimensional test, and the frequency of repeated experiments is 50.

The performance of the algorithms will be evaluated by the following aspects:

- in the case of a certain number of iterations, compare the accuracy (briefed as Perform.) of the optimal fitness value in each case. These values reflect the quality of the optimal solution obtained in the last;
- in the case of a certain number of iterations, compare the success rates (briefed as Prop.) which means that the algorithms achieve the accuracy (accepted error) that is defined in Table 2. These data reflect the stability of the algorithms;
- in the case of a certain number of iterations, compare the evolutionary trends of various algorithms, these figures reflect the evolution of the optimal solution in the evolutionary process.

### 4.2. Comparison between Random Topologies and Different Average Degrees

For the random topology, the first set of experiments used the canonical PSO algorithm to solve the five benchmark functions. By changing the *K* value, we generated random topologies with different average degrees for comparison and evaluation. Experimental results are shown in Table 3.

As can be seen from Table 3, with the increase in the value of *K*, the indicators of Perform. and Prop. have improved. For multimodal functions such as Schwefel, Schaffer's F6, Rastrigin, and Ackley function, with the increase in the value of *K*, when the *K* value is from 3 to 4 (i.e., the average degree of the random topology is between 5 and 7, substantially in about 6), the PSO algorithm could get better performance. And when the *K* value is larger, the performance is usually poor. For multimodal functions, when a particle has found a local optimal solution, if the interaction between particles is more, the dissemination of information will be very quickly, and the entire population is susceptible to rapid convergence to the local optimal solution. Therefore, too large average degree of population topology is not conducive to find the global optimal solution for a multimodal function.

Taking these factors together, when the value of *K* is at about 4, the PSO algorithm has a more satisfactory performance for most benchmark functions. Accordingly, the following experiments will generate random topologies which the *K* value is 4, and compare these topologies with other classic static population topologies. 

### 4.3. Comparison between Random Topologies and Classic Topologies

The second set of experiments used the DPPSO-Logistic algorithm to solve the five benchmark functions on the fully connected topology, ring topology, star topology, and random topology, wherein *K* value is set to 4 to generate random topologies. Comparison and evaluation are conducted by the evolutionary trends of algorithms with various population topologies. In the figures of evolutionary trends, different line types expressed different DPPSO-Logistic algorithms with various population topologies.

**Figure 2:**
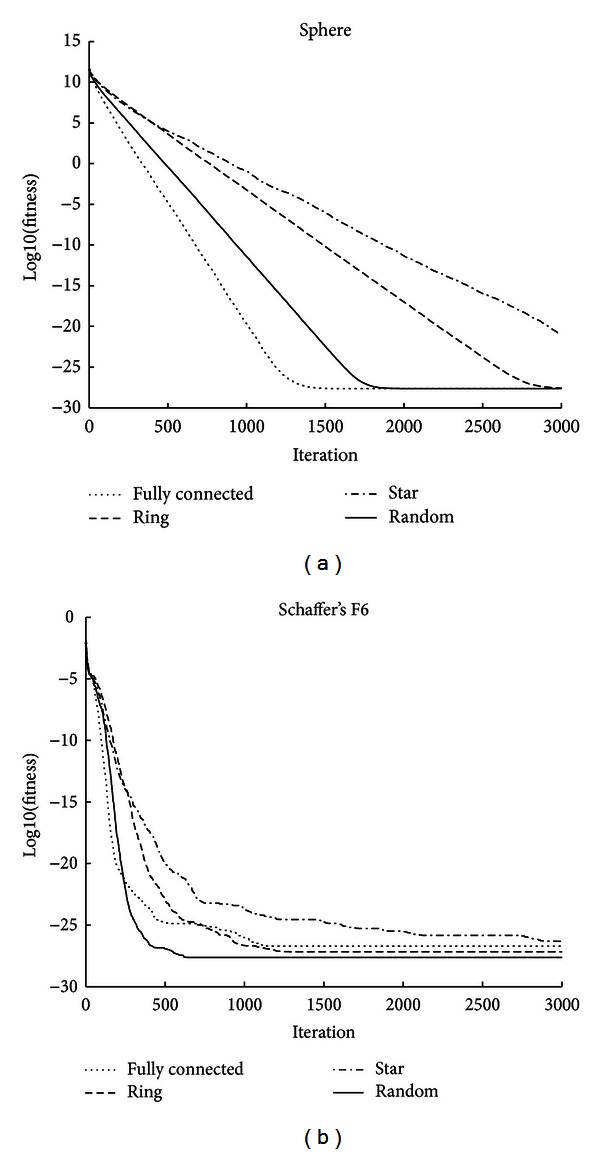
Comparison of evolutionary trend between four topologies (Sphere and Schaffer's F6).

For Sphere function (Figure 2), the performance of ring topology and star topology is poor, and the fully connected topology and random topology show better search ability. For Schaffer's F6 function (Figure 2), random topology is significantly better than the three classic neighborhood topologies.

**Figure 3:**
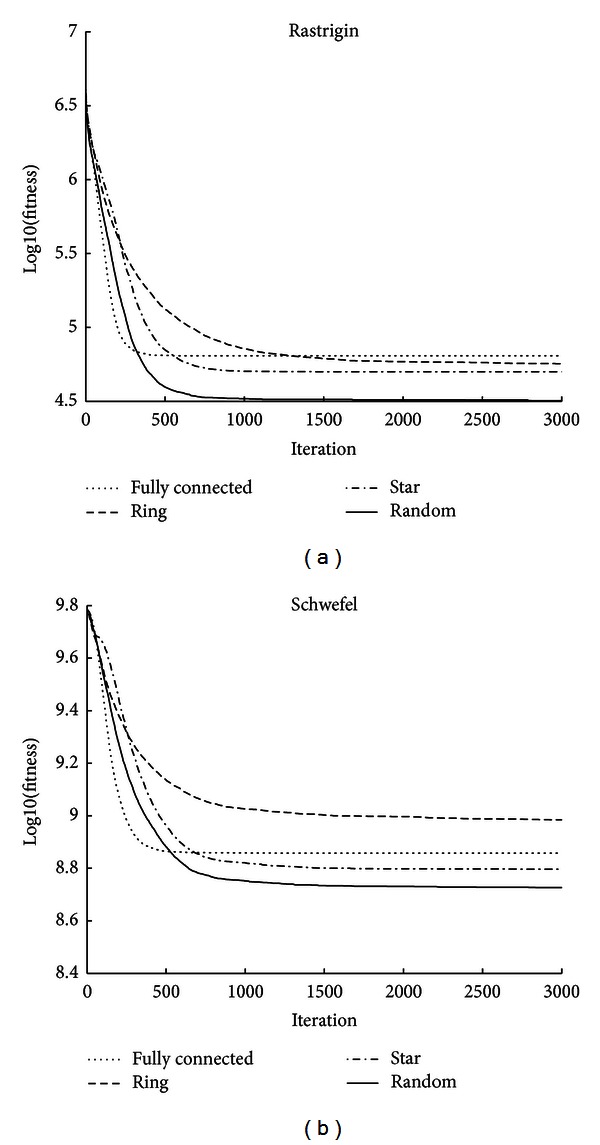
Comparison of evolutionary trend between four topologies (Rastrigin and Schwefel).

For Rastrigin function (Figure 3), random topology is superior to the three classic population topologies; in the early stages of evolution, the convergence speed of the random topology is almost the same as the fully connected topology; however, in the later stage of evolution, random topology shows a larger advantage, and the end result is better than the fully connected topology.

For Schwefel function (Figure 3), the performance of the ring topology is poor; the fully connected topology convergence fast in the early stage of evolution, but the end result is poor; the star topology has achieved good results; the convergence speed of random topology is second only to the fully connected topology in the early evolution stage, and the final result of random topology is better than the other classic topologies.

**Figure 4:**
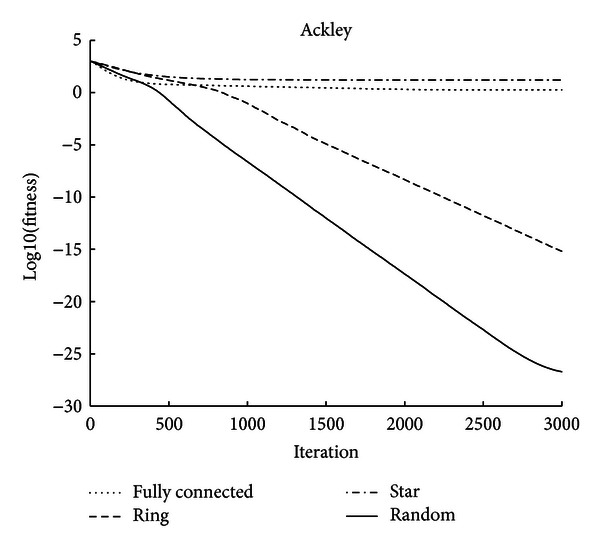
Comparison of evolutionary trend between four topologies (Ackley).

For Ackley function (Figure 4), the fully connected topology and the star topology show poor performance; the ring topology performs better; the random topology shows obvious advantage in both the convergence speed and the final result.

Overall, according to the convergence speed, random topology is relatively stable in the early stages of evolution, faster in the midstages of evolution, and shows a distinct advantage in the late stages of evolution. From the view of final result, the PSO algorithms using random topology demonstrate remarkable performance.

For unimodal function (such as Sphere function), because there is no problem of falling into a local optimum, the close ties between particles can make faster convergence and achieve better results. Therefore, the performance of the fully connected topology and random topology with a relatively high average degree is ideal. In the case of random topology for unimodal function, the convergence speed and the solution will be better with the greater average degree of population topology.

For multimodal function, it can be seen that the convergence speed will be faster when the average degree of population topology is increasing. If the average degree of population topology is too high, it is easy to fall into local optimum. On the average degree after 6, the optimal solution quality of most algorithms begins to decrease. When the average degree is between 5 and 7 (*K* is set to 4), the performance of algorithms is usually ideal.

## 5. Conclusion

 In this paper, we propose an improved method of generating random topology based on previous research. And we carry out in-depth research of the performance of the algorithms using random topology based on the canonical PSO and DPPSO-Logistic, respectively. Combined with experimental results, we conduct the analysis and interpretation of the performance of the algorithms from the perspective of graph theory. And empirical laws in generating random topologies are given according to our experimental results and theoretical analysis.

On the whole, relative to the three classic population topologies (fully connected topology, ring topology, and star topology), the algorithm has obvious advantages which is using the proposed random topology. Further work will include the theoretical analysis of different population topologies, as well as dynamic population topology strategy which is designed in accordance with the conclusions of this paper.

## Figures and Tables

**Algorithm 1:**
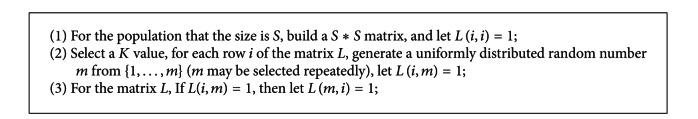
Clerc's generating method of random population topology.

**Algorithm 2:**
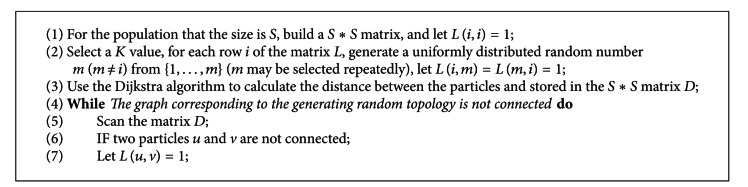
The proposed generating method of random population topology.

**Table 1:** Definition of benchmark functions.

Benchmark function	Formula
Sphere	$F(\vec{x})=\overset{n}{\underset{i=1}{\sum }}x_{i}^{2}$
Schaffer's F6	$F(\vec{x})=0.5+\frac{\left(\sin^{2}\sqrt{x^{2}+y^{2}}\right)-0.5}{{\left(1.0+0.001\left(x^{2}+y^{2}\right)\right)}^{2}}$
Rastrigin	$F(\vec{x})=10*n+\overset{n}{\underset{i=1}{\sum }}‍(x_{i}^{2}-10\cos(2\pi x_{i}))\;\;$
Schwefel	$F(\vec{x})=418.9829*n+\overset{n}{\underset{i=1}{\sum }}‍x_{i}\sin(\sqrt{\left|x_{i}\right|})$
Ackley	$F(\vec{x})=-20\cdot \exp\left(-0.2\sqrt{\frac{1}{n}\cdot \overset{n}{\underset{i=1}{\sum }}‍x_{i}^{2}}\right)-\exp\left(\frac{1}{n}\cdot \overset{n}{\underset{i=1}{\sum }}‍\cos\left(2\pi x_{i}\right)\right)+20+\exp(1)$

**Table 2:** Settings of benchmark functions.

Benchmark function	Dimension	Optimal value	Optimal solution	Range	Accepted error
Sphere	30	0	(0, 0, 0,…, 0)	(−100, 100)	0.01
Schaffer's F6	2	0	(0, 0)	(−100, 100)	0.00001
Rastrigin	30	0	(0, 0, 0,…, 0)	(−5.12, 5.12)	100
Schwefel	30	0	(0, 0, 0,…, 0)	(−500, 500)	6000
Ackley	30	0	(0, 0, 0,…, 0)	(−30, 30)	5

**Table 3:** Comparison between random topologies with different average degrees.

Benchmark function	*K*	Average degree	Clustering coefficient	Perform.	Prop.
Sphere	1	1.958	0.753	1.38*E* − 15	0.768507
	2	3.700	0.541	1.53*E* − 30	0.817693
	3	5.244	0.498	3.70*E* − 32	0.82624
	4	6.640	0.510	5.92*E* − 33	0.833993
	5	7.928	0.534	4.15*E* − 33	0.836973
	6	9.040	0.568	8.09*E* − 34	0.84178
	7	10.116	0.606	1.00*E* − 33	0.84702
	8	11.092	0.641	1.68*E* − 34	0.84278
	9	11.814	0.671	9.18*E* − 34	0.84446

Schaffer's F6	1	1.942	0.753	0.002915	0.497393
	2	3.700	0.545	0.001757	0.63916
	3	5.228	0.497	0.001749	0.681847
	4	6.626	0.508	0.00136	0.716053
	5	7.926	0.530	0.001943	0.6303
	6	8.998	0.563	0.003303	0.558613
	7	10.070	0.602	0.00272	0.59538
	8	11.078	0.643	0.003313	0.542707
	9	11.854	0.673	0.002915	0.558667

Rastrigin	1	1.956	0.751	63.65224	0.868587
	2	3.688	0.546	58.10571	0.923933
	3	5.198	0.500	58.64275	0.915993
	4	6.694	0.507	62.68228	0.934207
	5	7.940	0.532	58.80197	0.94144
	6	9.018	0.565	62.54299	0.93782
	7	10.118	0.608	59.10044	0.944673
	8	10.982	0.638	61.90622	0.908687
	9	11.916	0.674	63.51805	0.925493

Schwefel	1	1.956	0.752	4773.649	0.91388
	2	3.694	0.548	4366.848	0.942427
	3	5.234	0.500	4388.532	0.9436
	4	6.616	0.502	4399.952	0.943533
	5	7.922	0.532	4286.961	0.950633
	6	9.046	0.571	4397.724	0.950987
	7	10.074	0.602	4412.291	0.932327
	8	11.016	0.641	4420.19	0.951007
	9	11.734	0.667	4397.566	0.933313

Ackley	1	1.948	0.751	1.224299	0.960753
	2	3.690	0.549	1.030908	0.969733
	3	5.282	0.493	0.985613	0.972333
	4	6.672	0.507	1.256866	0.97304
	5	7.872	0.539	1.238158	0.973947
	6	9.028	0.568	1.593962	0.974087
	7	10.134	0.611	1.755415	0.97432
	8	10.922	0.640	1.919407	0.974847
	9	11.876	0.671	1.962931	0.97564
